# A new mutation of the *PCNT* gene in a Colombian patient with microcephalic osteodysplastic primordial dwarfism type II: a case report

**DOI:** 10.1186/1752-1947-8-191

**Published:** 2014-06-13

**Authors:** Harry Pachajoa, Felipe Ruiz-Botero, Carolina Isaza

**Affiliations:** 1 Faculty of Health, Universidad Icesi, Research Centre on Congenital Anomalies and Rare Diseases (CIACER), Calle 18 No. 122-135, bloque L, Oficina: 5025A Pance, Cali, Colombia; 2 Department of Morphology, Universidad Del Valle, Universidad del Valle Sede San Fernado, Calle 4B No 36-00, Area Morfología, Cali, Colombia

**Keywords:** Microcephalic osteodysplastic primordial dwarfism, Birth defects, Primordial dwarfism

## Abstract

**Introduction:**

Microcephalic osteodysplastic primordial dwarfism is a syndrome characterized by the presence of intrauterine growth restriction, post-natal growth deficiency and microcephaly. Microcephalic osteodysplastic primordial dwarfism type II is the most distinctive syndrome in this group of entities. Individuals affected by this disease present at an adult height of less than 100cm, a post-pubertal head circumference of 40cm or less, mild mental retardation, an outgoing personality and bone dysplasia.

**Case presentation:**

We report the first case of a five-year-old Colombian boy of mixed race ancestry (mestizo), with clinical features of microcephaly, prominent and narrow nose, arched palate, amelogenesis imperfecta, short stature, tall and narrow pelvis, disproportionate shortening of fore-arms and legs, and mild coxa vara. Analysis of the *PCNT* gene by sequencing showed the presence of a nucleotide change in exon 10, c. 1468C>T, evidencing a new mutation not reported in the literature for microcephalic osteodysplastic primordial dwarfism.

**Conclusion:**

The new mutation identified in this case could be associated with the severity of the phenotypic expression of the disease, resulting in the extreme short stature of the patient. Further studies are required to reach an explanation that can justify such findings, and it is vital to emphasize the importance of detection and follow-up by the epidemiological surveillance groups in birth defects and rare diseases.

## Introduction

Microcephalic osteodysplastic primordial dwarfism (MOPD) is a syndrome characterized by the presence of intrauterine growth restriction, post-natal growth deficiency, microcephaly and a similar phenotype to Seckel syndrome [1]. This condition was initially described by Majewski *et al*. [2], who characterized three distinct syndromes which he named microcephalic osteodysplastic primordial dwarfism types I, II and III. Majewski *et al*. also established the difference between these and Seckel syndrome, (a disorder that belongs to the primordial dwarfism group), due to the severity in the growth retardation, the presence of bone abnormalities and mild or absent mental retardation [3-7]. The first description of this syndrome likely corresponds to the siblings Manuel and Lucia Zarate. The latter lived between 1864 and 1890 and travelled in sideshows in England and throughout North America showcasing her unusual stature [8].

MOPD types I and III are considered variations of the same disorder, and are associated with skull, vertebra and limb dysplasia, brain malformations and premature death. Using homozygosity mapping of the whole genome to map the phenotype of MOPD I to chromosome 2q14.2, four different mutations were identified in the *RNU4ATAC* gene resulting in MOPD I, which was noted in the Amish population in Ohio, two German families and an Australian family of Maltese descent [9].

The MOPD type II (MOPD II, Mendelian Inheritance in Man (MIM) 210720) is a rare syndrome of autosomal recessive inheritance, and is the most distinctive of this group of pathologies. Affected individuals have an adult height under 100cm, a post-pubertal head circumference of 40cm or less, mild mental retardation, an outgoing personality, bone dysplasia, disproportionate shortening of fore-arms and legs, brachymetacarpia, "V" shaped redness of at least the distal femoral metaphysis, triangular distal femoral epiphysis, a high narrow pelvis, proximal femoral epiphysiolysis, coxa vara, abnormal dentition, increased risk of cerebrovascular disease, insulin resistance, and pre-mature fusion of the cranial sutures and fontanelles. MOPD II patients often present with pre-term birth, and their size *in utero* is occasionally confused with fetal intrauterine growth retardation [3,7].

A known genetic alteration has been described for MOPD II, a bi-allelic loss of function mutation in the *Pericentrin* gene (*PCNT*) located on chromosome 21. This gene has an important role in mitotic spindle organization; its loss prevents cell division resulting in severe growth retardation [10]; autosomical recessive inheritance has also been described [3].

We present the first report of a Colombian patient with MOPD type II, with a new mutation in the *PCNT* gene identified by sequencing.

## Case presentation

Our patient was the product of the first gestation of parents with common ancestry, both of them native to a small town located east of the department of Antioquia, Colombia. At gestation, the father was 36-years-old and the mother was 30 years of age.

The mother’s pre-natal history reports the ultrasound finding at 25 weeks of gestation to be of symmetrical intrauterine growth restriction without hemodynamic repercussion, ventriculomegaly, oligohydramnios and a single umbilical artery. Serial Doppler ultrasound follow-up performed at 35 weeks of gestation showed findings of fetal biometry below the third percentile for gestational age consistent with previous ultrasounds. At 28 weeks of gestation, a lung maturation scheme with corticosteroids was implemented, and at 36 weeks of gestation, a Cesarean section was scheduled due to premature rupture of the membranes with the fetal Non-Stress test reactive with decreased variability, no uterine activity and no progression of cervical changes.

A Cesarean section was performed without complications, producing a boy, weighing 1310g (<p3) with a length of 37cm (<p3). The boy presented with psychomotor developmental delay, crawled at 12 months of age, and walked at 17 months. At two years of age, he started growth hormone therapy, which was suspended a year later because of non-improvement.

**Figure 1:**
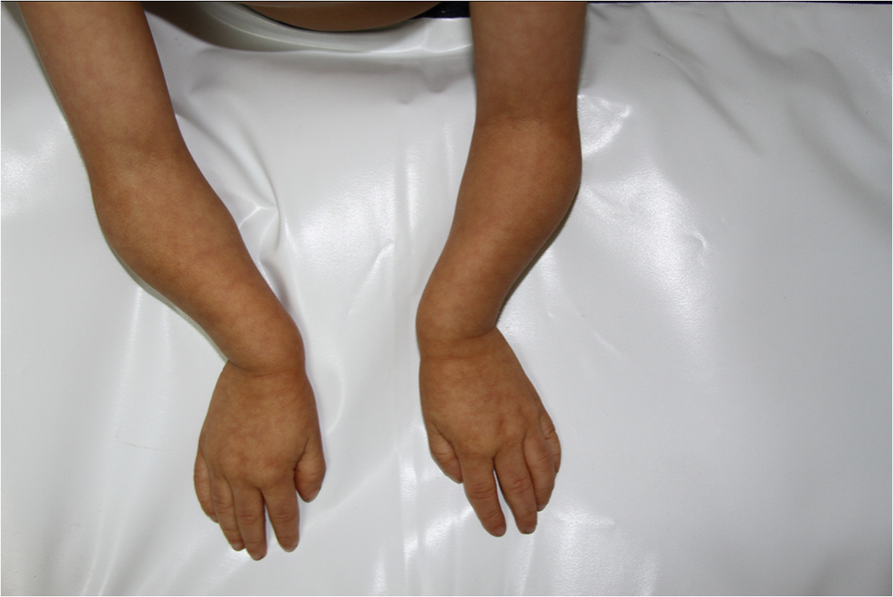
**Our patient at age 5.** Note the ulnar deviation of his hands.

Complementary studies performed when he was four years of age reported a computed tomography (CT) scan which identified IV ventricle and posterior fossa without alteration, microcephaly, closure of the metopic and sagittal structures, and also identified permeability of the coronal and lambdoid sutures. Total abdominal ultrasound and echocardiogram showed no abnormality.Our patient is currently five years of age with clinical findings that include microcephaly, downward slanting palpebral fissures, a prominent nose with a narrow nasal bridge, a convex nasal profile, prominent and narrow nasal tip, arched palate, amelogenesis imperfecta, ears with underdeveloped superior crus of the antihelix, an underdeveloped helix, bilateral clinodactyly, ulnar deviation, a high and narrow pelvis, fore-arms and legs disproportionately short, mild coxa vara, a sharp, high-pitched voice and a social personality (see Figure 1).

**Figure 2:**
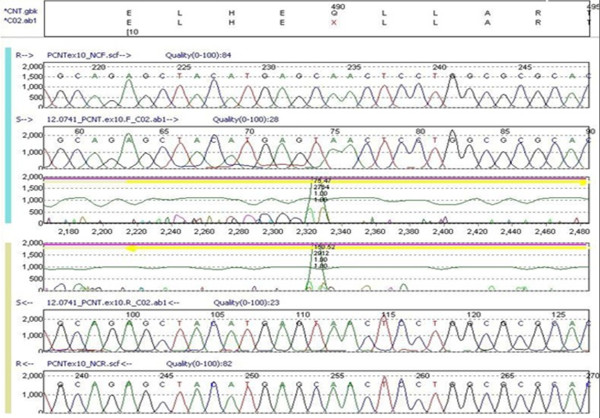
**Sequence analysis of the *PCNT *** **gene; note the presence of a nucleotide change in exon 10, c. 1468C>T, which results in the creation of a pre-mature stop codon.**

A *PCNT* gene mutation analysis was performed by polymerase chain reaction (PCR) amplification of all 47 coding exons of the *PCNT* gene. Products were sequenced in both forward and reverse directions from our patient's genomic deoxyribonucleic acid (DNA). The *PCNT* cDNA reference sequence used was NM_006031.5. DNA sequence analysis of the *PCNT* gene demonstrated the presence of a nucleotide change in exon 10, c. 1468C>T that results in the creation of a premature stop codon at amino acid position 490, p.Q490X, and is predicted to result in a truncated protein. The apparent homozygous c.1468C>T pathogenic sequence change could also be due to the presence of a deletion in one of our patient's *PCNT* alleles (that is, hemizygosity for the c.1468C>T pathogenic sequence change); however, since consanguinity or common ancestry is known to be present in this family, then homozygosity for this pathogenic sequence change is much more likely (see Figure 2).

We believe that due to the common ancestry found in our patient, homozygosity for this pathogenic sequence change is more probable.

## Discussion

The growth of an individual depends on the regulation of cell division and cell growth. Alterations in either of these regulatory pathways result in sub-somatic growth and contribute to the development of a wide variety of pathological conditions, including cancer and diabetes [11].

In 1967, Taybi and Linder described a form of congenital familiar dwarfism with cephalo-skeletal dysplasia; later, in 1982, three distinct types of MOPD were differentiated by Majewski based on clinical and radiological findings [2,6,12,13]. The three types have common findings: severe intrauterine and post-natal growth retardation, narrow forehead, prominent nose and micrognathia [3].

MOPD II differs from the other types by the presence of small dysplastic iliac wings, a high narrow pelvis, short and bowed humeri and femurs, metaphyseal widening, femoral epiphysiolysis, retarded epiphyseal maturation, elongated metaphysis and triangular epiphysis [3,14,15]. These clinical and radiographic findings are consistent with the clinical findings of our patient. Our patient is the product of the first gestation of parents with common ancestry, and has clinical features compatible with MOPD II, to whom is discovered a new *PCNT* gene mutation presenting a nucleotide change in exon 10, c. 1468C>T, which results in a premature stop codon at amino acid position 490, p.Q490X.

Rauch *et al*. located the alteration in chromosome 21q22.3, *PCNT2* gene locus, after mutational analysis of exon 47 of the *PCNT* gene in 25 unrelated patients with a clinical diagnosis of MOPD II. A total of 29 different null mutations were identified, and were compounded of 12 stop mutations and 17 frame changes (4 splice site mutations, 2 small insertions, 10 small selections and a deletion of the exon) scattered throughout the gene [10].

In patients with MOPD II, the *PCNT2* gene is transcribed, but not translated (absent or low protein levels), because its messenger ribonucleic acid (mRNA) is subject to decay mediated by a non-sense mRNA, directed by pre-translational surveillance mechanisms. Mutations in the *Pericentrine* gene (*PCNT*) cause primordial dwarfism [10].

Willems *et al*. identified 13 mutations in eight patients with MOPD II diagnosis and five patients with Seckel syndrome [4]. Consistent with reports such as Rauch *et al*.’s, the mutations were found distributed throughout the gene. Of the identified mutations, the c.3109G/T (exon 15) mutation was previously reported by Rauch *et al*. [10]. It is noteworthy that the patients affected by this mutation were both from Turkey. Willems *et al*. concluded that all identified mutations correspond to loss of function mutations, and genetic homogeneity was confirmed for this pathology [4]. In a retrospective analysis of this study, five patients diagnosed as having Seckel syndrome corresponded to the spectrum of MOPD II but had been excluded because of mental retardation and a height greater than 110cm. It is suggested that all patients with a diagnosis of Seckel syndrome must be reassessed for a possible wrong diagnosis [4].

Of importance is that our patient, compared to those reported by Rauch *et al*. [10] and Willems *et al*. [4], presents a nucleotide change resulting in the creation of a premature stop codon that is predicted to result in a truncated protein, information that is consistent with previous reports. Due to the common ancestry of our patient's parents, it is highly likely homozygosity is the cause of this pathological change of sequence [4,10].

It should be noted that when we compare the height of the reported patient with the growth charts for patients with MOPD II proposed by Bober *et al*. [1], the patient is well below -2 standard deviations; this leaves the possibility that this difference occurs as a consequence of the origin and population group of the patient east of Antioquia (Colombia), unlike the sample of patients used by Bober *et al*., which belong to the registry of patients with MOPD II at the Nemours/Alfred I. duPont Hospital for Children in Wilmington, DE, USA (see Table 1) [1].

**Table 1:** **Anthropometric follow-up compared to the growth charts for patients with microcephalic osteodysplastic primordial dwarfism type II proposed by Bober *et al*. 2012**[1]

**Age**	**Head circumference**	*SD	**Weight**	*SD	**Height**	*SD	**Internal intercanthal distance**	**External intercanthal distance**	**Interpupillary distance**
Birth (36 weeks of gestation)	-	-	1.3kg	Mean and 1 SD	37cm	-1 SD and mean	-	-	-
4 years 5 months	41cm	1 SD - 2 SD	5.9kg	Mean and 1 SD	71cm	<-2 SD	-	-	-
4 years 9 months	41cm	2 SD - 2 SD	6.1kg	Mean and 1 SD	72cm	<-2 SD	-	-	-
4 years 11 months	41cm	3 SD - 2 SD	6.1kg	Mean and 1 SD	72cm	<-2 SD	-	-	-
5 years 1 month	41.2cm	4 SD - 2 SD	6.5kg	Mean and 1 SD	72cm	<-2 SD	2.5cm	7.5cm	4.5cm
5 years 3 months	41.2cm	5 SD - 2 SD	6kg	Mean and 1 SD	72cm	<-2 SD	2.5cm	7.5cm	4.5cm
5 years 7 months	41.2cm	6 SD - 2 SD	6.7kg	Mean and 1 SD	72cm	<-2 SD	2.5cm	7.5cm	4.5cm

*Ref. Bober *et al*. 2012 [1]. SD, standard deviation.

Another possibility which may explain this finding corresponds to the phenotypic expression of the identified mutation. This leads us to assume that although mutations in the *PCNT* gene are associated with the appearance of primordial dwarfism, the type of mutation or site of the alteration are associated with the severity of the phenotypic expression of the disease, causing the extreme short stature of our patient, even when compared to patients of the same age group diagnosed with MOPD II.

## Conclusion

In conclusion, further studies are required to reach an explanation that can justify such findings and it is vital to emphasize the importance of detection and follow-up by the epidemiological surveillance groups in birth defects and rare diseases. The development of new information may lead to the solution of the many unknowns in the understanding of the pathologies belonging to the group of primordial dwarfism, at a genetic and patho-physiological level. Additionally, it is necessary to study the population group in eastern Antioquia due to the existence of other patients with clinical manifestations similar to those of MOPD II in that community.

## Consent

Written informed consent was obtained from our patient’s legal guardian for publication of this case report and any accompanying images. A copy of the written consent is available for review by the Editor-in-Chief of this journal.

## Abbreviations

MOPD: Microcephalic osteodysplastic primordial dwarfism; cDNA: Complementary deoxyribonucleic acid; CT: Computed tomography; PCR: Polymerase chain reaction; mRNA: Messenger Ribonucleic acid; *PCNT*: *Pericentrine* gene.

## Competing interests

The authors declare that they have no competing interests.

## Authors’ contributions

HP, FR and CI analyzed and interpreted the patient data and wrote the manuscript. All authors have read and approved the final manuscript.
